# Surveillance of Dihydropteroate Synthase Genes in *Stenotrophomonas maltophilia* by LAMP: Implications for Infection Control and Initial Therapy

**DOI:** 10.3389/fmicb.2016.01723

**Published:** 2016-10-26

**Authors:** Jin Zhao, Yubin Xing, Wei Liu, Wentao Ni, Chuanqi Wei, Rui Wang, Yunxi Liu, Youning Liu

**Affiliations:** ^1^Department of Respiratory Diseases, Chinese PLA General HospitalBeijing, China; ^2^Department of Infection Management and Disease Control, Chinese PLA General HospitalBeijing, China; ^3^Testing Center of HMI, Chinese PLA General HospitalBeijing, China; ^4^Department of Clinical Pharmacology, Chinese PLA General HospitalBeijing, China

**Keywords:** loop-mediated isothermal amplification, LAMP, *Stenotrophomonas maltophilia*, dihydropteroate synthase, *sul* gene, sulfamethoxazole, minocycline

## Abstract

*Stenotrophomonas maltophilia* is a common nosocomial pathogen that causes high morbidity and mortality. Because of its inherent extended antibiotic resistance, therapeutic options for *S. maltophilia* are limited, and sulfamethoxazole/trimethoprim (SXT) is the only first-line antimicrobial recommended. However, with the spread of dihydropteroate synthase (*sul1* and *sul2*) genes, global emergence of SXT resistance has been reported. There is an urgent need to develop a rapid and sensitive but cost-efficient method to monitor the dissemination of *sul* genes. In this study, we developed loop-mediated isothermal amplification (LAMP) assays for *sul1* and *sul2* using real-time turbidity and hydroxy naphthol blue coloration methods. The assays could quickly detect *sul* genes with high sensitivity and specificity. The LAMP detection limit was 0.74 pg/reaction of extracted genomic DNA for *sul1* and 2.6 pg/reaction for *sul2*, which were both 10-fold more sensitive than the corresponding traditional PCR assays. Additionally, the LAMP assays could positively amplify DNA from *sul1*-producing strains, but not from the negative controls. We then used the LAMP assays to investigate the dissemination of *sul* genes among *S. maltophilia* isolates from patients in three hospitals in Beijing, China. Among 450 non-duplicated samples collected during 2012–2014, 56 (12.4%) strains were SXT-resistant. All these SXT-resistant strains were positive for *sul* genes, with 35 (62.5%) carrying *sul1*, 17 (30.4%) carrying *sul2*, and 4 (7.1%) carrying both *sul1* and *sul2*, which indicated that *sul* genes were the predominant resistance mechanism. Of 394 SXT-susceptible strains, 16 were also *sul*-positive. To provide epidemiological data for the appropriate choice of antimicrobials for treatment of *sul*-positive *S. maltophilia*, we further tested the susceptibility to 18 antimicrobials. Among these, *sul*-positive strains showed the highest susceptibility to tetracycline derivatives, especially minocycline (MIC_50_/MIC_90_, 0.5/4; susceptibility rate, 95.4%). Ticarcillin-clavulanate and new fluoroquinolones (moxifloxacin and levofloxacin) also showed some *in vitro* activity. Apart from these three kinds of antimicrobials, other agents showed poor activity against *sul*-positive strains.

## Introduction

*Stenotrophomonas maltophilia* is the third most common non-fermentative gram-negative bacillus isolated from hospitals and has emerged as an important opportunistic pathogen (Looney et al., 2009). It is associated with a broad range of serious human infections, including pneumonia, bloodstream infections, and urinary tract infections, especially in immunocompromised patients (Looney et al., 2009; Chang et al., 2015). An increasing incidence and considerable morbidity have been reported in recent decades (Falagas et al., 2009; Looney et al., 2009). Since *S. maltophilia* shows a high level of intrinsic resistance to most commonly used antimicrobial agents, including most cephalosporins, carbapenems, aminoglycosides, and quinolones, its treatment is extremely difficult. Sulfamethoxazole/trimethoprim (SXT) is the only first-line antimicrobial recommended for treatment because of its good antibacterial activity *in vitro* and low incidence of resistance (Abbott et al., 2011; Wu et al., 2012; Wei et al., 2015). Although other mechanisms, such as *dfrA* and *SmeDEF* genes, might influence the minimal inhibitory concentration (MIC), the *sul* genes have generally been recognized as the predominant resistance mechanism (Toleman et al., 2007; Hu et al., 2011). The *sul* genes are usually located on mobile genetic elements, and can mobilize horizontally and vertically, assisted by the spread of class 1 integrons and insertion sequence common region (ISCR) elements. The global emergence of SXT resistance mediated by the dissemination of *sul* genes has been reported (Toleman et al., 2007). There is an urgent need to develop a rapid, simple, and cost-effective assay for the epidemiological investigation and surveillance of the dissemination of *sul* genes, which could provide epidemiological data to guide the establishment of infection control measures and the choice of initial antimicrobials. Loop-mediated isothermal amplification (LAMP) is a novel DNA amplification method. It was first developed in the year 2000 and can efficiently and specifically amplify DNA under isothermic conditions within 60 min using *Bst* DNA polymerase (Notomi et al., 2000; Dong et al., 2015a). The only requirement to perform LAMP assays is having access to a constant-temperature apparatus. Moreover, by adding hydroxy naphthol blue (HNB), the results can be evaluated by the naked eye, thus avoiding the process of agarose gel electrophoresis that is required for traditional PCR. LAMP assays have been used for the rapid diagnosis of infectious diseases and the surveillance of epidemics of bacteria, viruses, and parasites (Sun et al., 2010; Wang et al., 2011; Soo et al., 2013). Recently, the technique has also been used for the surveillance of genes encoding various carbapenemase enzymes (Qi et al., 2012; Nakano et al., 2015; Yamamoto et al., 2015; Kim et al., 2016), but there have been no reports on the use of LAMP for the detection of SXT-resistance genes.

The objectives of this study were to establish a rapid, simple, and cost-effective assay for the detection of *sul* genes in *S. maltophilia* and to investigate the dissemination and antimicrobial susceptibility of *S. maltophilia* carrying *sul* genes. These data are essential to guide the establishment of infection control measures and the choice of an appropriate initial antibacterial agent. First, we developed LAMP assays for the detection of *sul1* and *sul2* and confirmed their specificity and sensitivity using two different detection methods, namely sample turbidity and HNB coloration. We then used the LAMP assays to study the dissemination of *sul* genes in three hospitals in Beijing, China during 2012–2014. Finally, the susceptibility of the *sul*-positive *S. maltophilia* strains to 18 commonly used antimicrobial agents was investigated.

## Materials and Methods

### Bacterial Isolation and Identification

In this study, during 2012–2014, 450 non-duplicated clinical *S. maltophilia* isolates were collected from hospitalized patients in the Chinese PLA General Hospital, Peking Union Medical College Hospital, and Air Force General Hospital in Beijing, China. Bacterial species identification was performed using a Vitek^®^ II bacterial identification system (bioMérieux, Marcy-l’Étoile, France) and further confirmed by species-specific PCR (Whitby et al., 2000). Three *sul1* (X2, P121, and K106) and *sul2* (X133, P57, and K14) positive control DNAs were used in the establishment of the LAMP assays and were confirmed to carry *sul* genes by PCR and sequencing of the PCR products. *S. maltophilia* ATCC 13637, K279a, and P129 were used as negative controls for estimating the specificity of the LAMP assays. Whole genome sequencing of these strains has been performed and they were confirmed to carry genes encoding multidrug-efflux pumps, β-lactamases, and aminoglycoside-modifying enzymes, but they did not carry *sul* genes (Crossman et al., 2008; Davenport et al., 2014).

The research protocol was approved by the Ethics Committee of Chinese PLA General Hospital, Peking Union Medical College Hospital, and Air Force General Hospital, and written informed consent was obtained from patients. Our research protocol did not affect patients’ health, safety, or privacy.

### PCR Detection of *sul* Genes

Whole genomic DNA (including plasmid DNA) was extracted using the protocol described by a previous study (Yang et al., 2014). The bacterial strains were cultured in brain–heart infusion broth at 37°C. Genomic DNA was extracted using a Wizard^®^ Genomic DNA Purification Kit (Promega, Madison, WI, USA) according to the manufacturer’s recommended protocol. The extracted DNA was used as a template in the LAMP and PCR reactions.

To compare the sensitivity of LAMP assays and traditional PCR, *sul1* detection was performed using the PCR primers shown in **Table 1**. The target products were 250 bp for *sul1* and 276 bp for *sul2*. The PCR cycling parameters were: initial PCR activation, 95°C for 5 min; amplification, 30 cycles of 95°C for 30 s, 55°C for 30 s, and 72°C for 30 s; final extension step, 72°C for 10 min. A 25-μl reaction volume was used, and the products were separated by 1% (w/v) agarose (Amresco, Solon, OH, USA) gel electrophoresis and stained with ethidium bromide. Images were documented with a Gel Doc^TM^ EQ imaging system (Bio-Rad, Hercules, CA, USA).

**Table 1:** Primers used for the specific amplification of the *sul1* and *sul2* genes.

Target gene	Reaction type	Primer	Sequence (5′–3′)	Location
*sul1*	PCR	SUL1-F	GCTATTGGTCTCGGTGTCGC	612–631
		SUL1-B	GCATGATCTAACCCTCGGTCT	836–816
*sul2*	PCR	SUL2-F	TTTCGGCATCGTCAACATAA	21–40
		SUL2-B	CCACGCGACAAGGCATA	296–280
*sul1*	LAMP	Sul1-33F3	GGCCGATGAGATCAGACGT	174–192
		Sul1-33B3	TCCCGCTGCGCTGAGT	392–377
		Sul1-33FIP	TGGGTTTCCGGTTGGAAGCTGT, CCGCTCTTAGACGCCCT	266–245, 199–215
		Sul1-33BIP	TCCAAGGATTTCCTGACCCTGC, GCATAACCACCAGCCTGCA	308–329, 376–358
		Sul1-33LF	AAACACGGTGCATCTGATCG	238–219
		Sul1-33LB	GCTCTATCCCGATATTGCTGAGG	330–352
*sul2*	LAMP	Sul2-121F3	ACCCGCTGGCGACATC	423–438
		Sul2-121B3	AGAAGCACCGGCAAATCG	623–606
		Sul2-121FIP	TGATACCGGCACCCGTCAGC, ATGGATCACATTGCGGCGTT	499–480, 439–458
		Sul2-121BIP	CTTGATCCCGGCATGGGGTTTT, GCCGCAATTCATCGAACCG	517–538, 598–580
		Sul2-121LF	CGATGCGCGCGTCAAAG	475–459
		Sul2-121LB	GGCTGCTCCCGAAACCT	546–562

*The sequences CP011493 (80815–81654) and CP009257 (2540172–2540987) obtained from the NCBI GenBank database were used for the design of sul1-specific and sul2-specific PCR and LAMP primers, respectively. F3, forward outer primer; B3, backward outer primer; FIP, forward internal primer; BIP, backward internal primer; LF, loop forward primer; LB, loop backward primer.*

### LAMP Reaction

The sequences CP011493 (80815–81654) and CP009257 (2540172–2540987) obtained from the NCBI GenBank database were used for the design of *sul1*-specific and *sul2*-specific PCR and LAMP primers, respectively. The primers were designed and analyzed by PrimerExplorer software^1^. The sequences and locations of primers for *sul1* and *sul2* are both shown in **Table 1**.

The LAMP reactions were performed in a final volume of 25 μl containing 12.5 μl reaction mixture, 1 μl *Bst* DNA polymerase, and 2 μl template using the Loopamp DNA Amplification Kit (Loopamp DNA Amplification Kit; Eiken Chemical, Co., Ltd, Tochigi, Japan) for real-time turbidimetry or with the further addition of 1 μl HNB solution (Eiken Chemical, Co., Ltd) for visual detection. Primers were used at the concentrations of 1.6 μM for the forward internal primer and backward internal primer, 0.8 μM for the loop forward primer and loop backward primer, and 0.2 μM for the forward outer primer and backward outer primer. The reactions were performed in the reaction tubes (Eiken Chemical, Co., Ltd) for 60 min at 65°C.

Two different methods, namely sample turbidity and HNB coloration, were used to detect the LAMP products. Real-time changes in turbidity were monitored by spectrophotometric analysis by recording the optical density (650 nm) every 6 s with a Loopamp Realtime Turbidimeter (LA-320c; Eiken Chemical, Co., Ltd). For direct visual detection, 1 μl of HNB detection reagent was added to the reaction. The amount of DNA template used in the LAMP reaction mixture was equal to that used in PCR reactions. Each experiment was performed at least three times.

### Antimicrobial Susceptibility Testing

Eighteen clinically used antibacterial agents were tested in our study, as follows: SXT, ceftazidime, minocycline, levofloxacin, chloramphenicol, ticarcillin/clavulanate, tigecycline, doxycycline, moxifloxacin, cefepime, cefoperazone/sulbactam, meropenem, amikacin, colistin, fosfomycin, aztreonam, azithromycin, and rifampicin. Tigecycline was obtained from Wyeth Pharmaceutical (Wyeth Pharmaceutical, Philadelphia, PA, USA); moxifloxacin was obtained from Bayer Healthcare (Bayer Pharma, Wuppertal, AG, Germany); Colistin was purchased from Sigma-Aldrich (St. Louis, MO, USA); other antimicrobial agents were purchased from the National Institute for the Control of Pharmaceutical and Biological Products (NICPBP, Beijing, China). The antimicrobial powders were used to prepare stock solutions, as described by the Clinical and Laboratory Standards Institute (CLSI, 2015). MICs of the *sul*-positive strains were determined for each isolate by the Mueller–Hinton agar (Difco, Franklin Lakes, NJ, USA) macrodilution method. *Pseudomonas aeruginosa* ATCC 27853, *Staphylococcus aureus* ATCC 29213, and *Escherichia coli* ATCC 25922 were used as quality control strains to ensure the accuracy and reliability of the assays. Interpretive breakpoints for susceptibility are available only for SXT, ceftazidime, minocycline, levofloxacin, chloramphenicol, and ticarcillin/clavulanate (CLSI, 2015). Other antimicrobial agents with no published breakpoint criteria for *S. maltophilia* were interpreted with reference to those for *Acinetobacter* spp. or Enterobacteriaceae published by CLSI (2015) (**Table 3**).

## Results

### Establishment of LAMP Assays for the Detection of *sul1* and *sul2*

**FIGURE 1:**
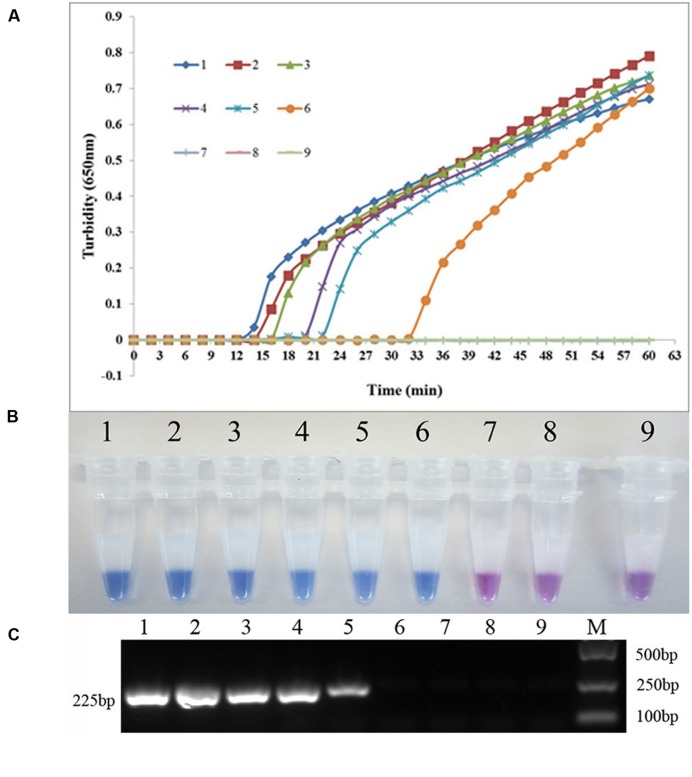
**Comparison of the sensitivities of LAMP and conventional PCR assays for detecting *sul1* in *Stenotrophomonas maltophilia*. (A)** Turbidity was monitored every 6 s using a Loopamp Realtime Turbidimeter with detection at 650 nm. The reaction was performed at 65°C. **(B)** The results were visualized by adding hydroxy naphthol blue (HNB) to the reaction mixture before the reaction. **(C)** PCR products were separated by 1% (w/v) agarose gel electrophoresis and stained with ethidium bromide. (1) 74.0 ng; (2) 7.4 ng; (3) 0.74 ng; (4) 74.0 pg; (5) 7.4 pg; (6) 0.74 pg; (7) 74.0 fg; (8) 7.4 fg; (9) blank control (double-distilled water).

**FIGURE 2:**
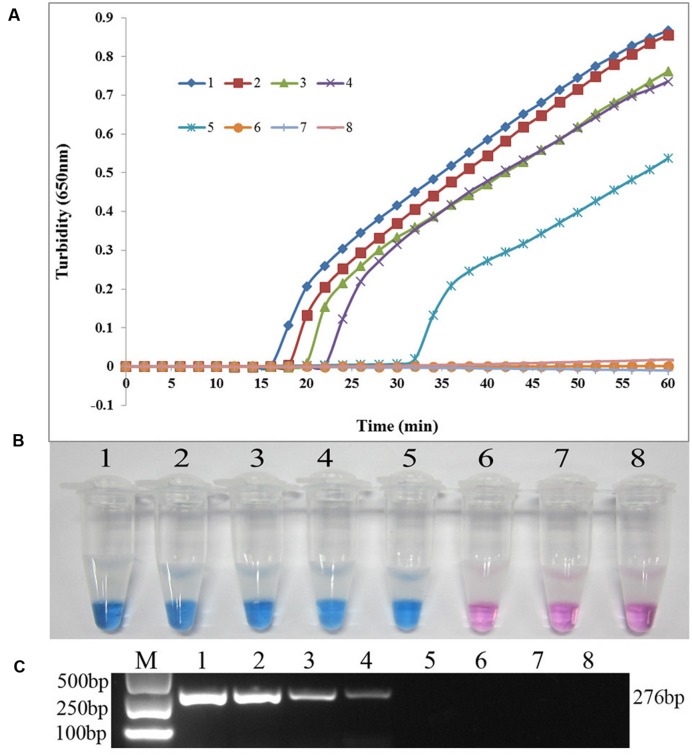
**Comparison of the sensitivities of LAMP and conventional PCR assays for detecting *sul2* in *S. maltophilia*. (A)** Turbidity was monitored every 6 s using a Loopamp Realtime Turbidimeter with detection at 650 nm. The reaction was performed at 65°C. **(B)** The results were visualized by adding HNB to the reaction mixture before the reaction. **(C)** PCR products were separated by 1% (w/v) agarose gel electrophoresis and stained with ethidium bromide. (1) 26.0 ng; (2) 2.6 ng; (3) 0.26 ng; (4) 26.0 pg; (5) 2.6 pg; (6) 0.26 pg; (7) 26.0 fg; (8) blank control (double-distilled water).

The *sul1*-specific and *sul2*-specific LAMP primers were designed and analyzed using PrimerExplorer software (**Table 1**). To compare the detection limit of the LAMP method with that of the conventional PCR method, genomic DNA was extracted from *S. maltophilia* X2 and serially diluted 10 times from 37.0 ng/μL to 3.7 fg/μL. As shown in **Figure 1**, the detection limits of the real-time turbidity and HNB methods were 0.74 pg/reaction of extracted whole genomic DNA, 10-fold more sensitive than that of the PCR assay (7.4 pg/reaction) (**Figure 1**). Similarly, the detection limit of LAMP specific to *sul2* was 2.6 pg/reaction, which is 10 times more sensitive than that obtained for the conventional PCR method (**Figure 2**).

**FIGURE 3:**
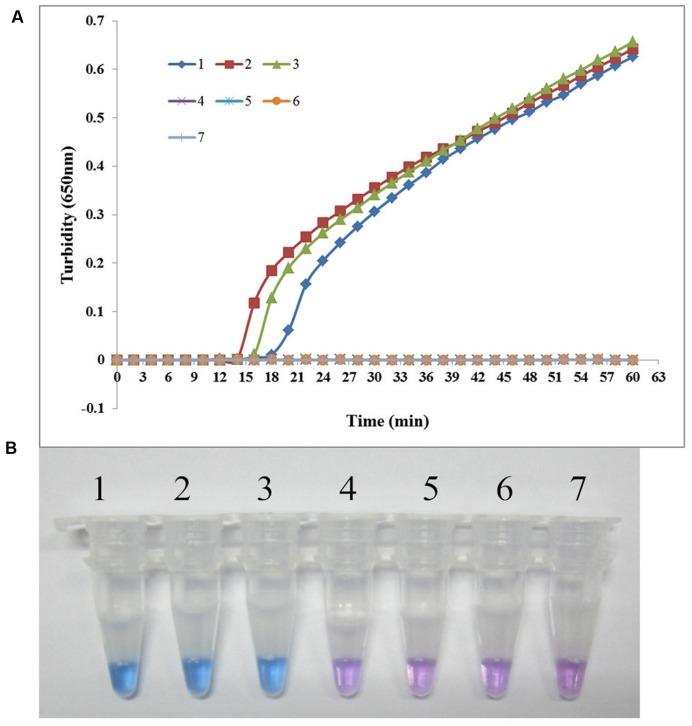
**Specificity of the LAMP reaction for detecting *sul1* in *S. maltophilia*. (A)** Turbidity was monitored every 6 s using a Loopamp Realtime Turbidimeter with detection at 650 nm. The reaction was performed at 65°C. **(B)** The results were visualized by adding HNB to the reaction mixture before the reaction. (1) X2 (positive control for *sul1* from Peking Union Medical College Hospital); (2) P121 (positive control for *sul1* from Chinese PLA General Hospital); (3) K106 (positive control for *sul1* from Chinese PLA General Hospital); (4) *S. maltophilia* K279a; (5) *S. maltophilia* ATCC 13637; (6) P129 (negative control for *sul* from Chinese PLA General Hospital, confirmed by genome sequencing); (7) double-distilled water.

**FIGURE 4:**
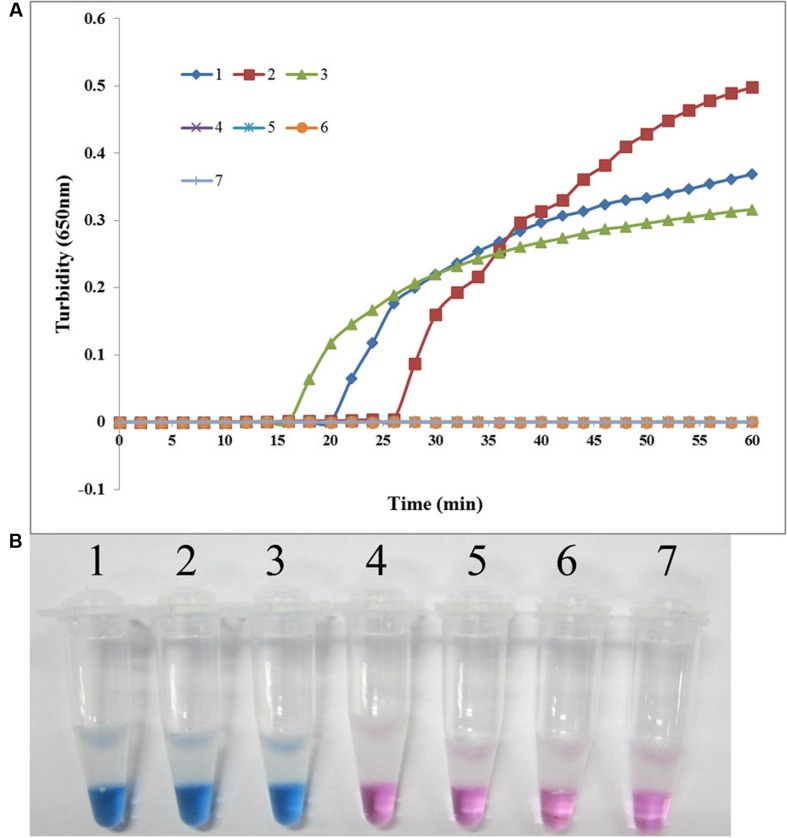
**Specificity of the LAMP reaction for detecting *sul2* in *S. maltophilia.* (A)** Turbidity was monitored every 6 s using a Loopamp Realtime Turbidimeter with detection at 650 nm. The reaction was performed at 65°C. **(B)** The results were visualized by adding HNB to the reaction mixture before the reaction. (1) X133 (positive control for *sul2* from Peking Union Medical College Hospital); (2) P57 (positive control for *sul2* from Chinese PLA General Hospital); (3) K14 (positive control for *sul2* from Chinese PLA General Hospital); (4) *S. maltophilia* K279a; (5) *S. maltophilia* ATCC 13637; (6) P129 (negative control for *sul* from Chinese PLA General Hospital, confirmed by genome sequencing); (7) double-distilled water.

The specificity of the LAMP assay for detecting *sul1* was evaluated using X2, P121, and K106 carrying *sul1* as positive controls, *S. maltophilia* ATCC 13637, K279a, and P129 as negative controls, and distilled water as blank control. As shown in **Figure 3**, both methods positively amplified DNA from *sul1*-producing strains, but not from the negative and blank controls. The results showed that the assay was highly specific for detecting *sul1*. The same results were observed for the developed *sul2* LAMP assay (**Figure 4**).

### Dissemination of *sul* Genes in *S. maltophilia*

During 2012–2014, 450 non-duplicated *S. maltophilia* strains were collected in our study, and 56 (12.4%) strains were resistant to SXT. As shown in **Table 2**, all these 56 SXT-resistant strains were tested positive for *sul* genes, among which 35 (62.5) strains carried *sul1*, 17 (30.4%) strains carried *sul2*, and 4 (7.1%) strains carried both *sul1* and *sul2*. Among 394 SXT-susceptible strains, 16 (4.1%) carried *sul* genes.

**Table 2:** Detection rates of *sul* genes in *Stenotrophomonas maltophilia* isolated from China during 2012–2014.

*sul* gene(s)	Detection rate (%)		
	SR-SMA (*n* = 56)	SS-SMA (*n* = 394)	Total (*n* = 450)
*sul1*	35 (62.5)	12 (3.0%)	47 (10.4)
*sul2*	17 (30.4)	4 (1.0)	21 (4.7)
*sul1*+*sul2*	4 (7.1)	0	4 (0.9)
total	56 (100)	16 (4.1)	72 (16)

*SR-SMA, sulfamethoxazole/trimethoprim-resistant S. maltophilia; SS-SMA, sulfamethoxazole/trimethoprim-susceptible S. maltophilia.*

### Antimicrobial Susceptibility of *sul*-Positive *S. maltophilia*

Among 18 clinically used antimicrobials, the susceptibility rates of *S. maltophilia* were 95.4% for minocycline, 68.1% for doxycycline and tigecycline, 50.0% and 59.7% for levofloxacin and moxifloxacin, respectively, and 69.4% for ticarcillin-clavulanate. The susceptibility rates for 12 other antimicrobial agents were less than 50%, while for the agents meropenem, amikacin, colistin, ceftazidime, chloramphenicol, aztreonam, sulfamethoxazole/trimethoprim, azithromycin, and rifampicin, *S. maltophilia* had resistance rates exceeding 70% (**Table 3**).

**Table 3:** *In vitro* susceptibility of 72 *sul*-positive *S. maltophilia* strains to 18 clinically used antimicrobials.

Antimicrobial agents	MIC (mg/L)			Susceptibility (%)		
	Range	MIC_50_	MIC_90_	S	I	R
SXT	19/1 to >304/16	152/8	>304/16	22.2		77.8
MIN	0.25–16	0.5	4	95.4	2.8	1.4
LEV	0.5–32	2	32	50	6.9	43.1
CHL	2 to >64	64	>64	5.6	12.5	81.9
CET	2 to >64	64	>64	23.6	5.6	70.8
T-C	1 to >128	8	128	69.4	13.9	16.7
TIG^†^	0.5–32	2	8	68.1	12.5	19.4
DOX^∗^	1–64	4	16	68.1	15.3	16.7
MOX^§^	0.25–32	2	16	59.7	6.9	33.3
CEP^†^	2–128	32	64	18.1		81.9
C-S^†^	4 to >256	32	128	40.3	18.1	41.7
MEP^∗^	32 to >32	>32	>32	0	0	100
AMK^∗^	32 to >64	>64	>64	0	2.8	97.2
COL^∗^	1 to >64	16	>64	12.5		87.5
FOS^†^	32 to >256	128	256	22.2	47.2	30.6
AZT^†^	16 to >32	>32	>32	0	0	100
AZM^†^	32 to >64	64	>64	0	0	100
RFP^#^	2–64	16	32	0	0	100

*SXT, sulfamethoxazole/trimethoprim; MIN, minocycline; LEV, levofloxacin; CHL, chloramphenicol; CET, ceftazidime; T-C, ticarcillin-clavulanate; TIG, tigecycline; DOX, doxycycline; MOX, moxifloxacin; CEP, cefepime; C-S, cefoperazone/sulbactam; MEP, meropenem; AMK, amikacin; COL, colistin; FOS, fosfomycin; AZT, aztreonam; AZM, azithromycin; RFP, rifampin. ^∗^MIC breakpoint criteria of these antimicrobials for S. maltophilia refer to susceptible breakpoint criteria for Acinetobacter spp. published by CLSI (2015); ^†^MIC breakpoint criteria of these antimicrobials for S. maltophilia refer to susceptible breakpoint criteria for Enterobacteriaceae published by CLSI (2015); ^§^ MIC breakpoint criteria of MOX for S. maltophilia refer to susceptible breakpoint criteria for anaerobes published by CLSI (2015); ^#^MIC breakpoint criteria of RFP for S. maltophilia refer to susceptible breakpoint criteria for Staphylococcus spp. published by CLSI (2015).*

## Discussion

*Stenotrophomonas maltophilia* is an opportunistic pathogen, mainly causing pneumonia and bloodstream infections in immunocompromised patients, with high morbidity and mortality (Falagas et al., 2009; Looney et al., 2009; Chang et al., 2015). Because of its intrinsic and acquired resistance to most commonly clinical used antibiotics, its treatment is extremely difficult, and SXT is the only first-line drug recommended (Looney et al., 2009; Chang et al., 2015), with an overall susceptibility rate of higher than 90% (Abbott et al., 2011; Wu et al., 2012; Chang et al., 2015; Wei et al., 2015). Resistance to SXT in *S. maltophilia* will lead to more treatment failure and higher mortality, and the global emergence of an increasing tendency toward SXT resistance mediated by the dissemination of *sul* genes has been reported. Surveillance of the dissemination of *sul* genes by rapid and cost-efficient LAMP assays could contribute to the control of SXT resistance and the reduction of *S. maltophilia* infection incidence. In the current study, we designed primers specific for both *sul1* and *sul2* for use in LAMP assays to monitor the prevalence of *sul* genes among clinical isolates of *S. maltophilia*. The LAMP detection method has several advantages over traditional PCR as a tool for use in epidemiological surveys. First, LAMP is generally less time- and labor-intensive, and it proceeds under an isothermal condition within 60 min (Notomi et al., 2000; Dong et al., 2015a; Li et al., 2015). Second, LAMP is more cost-efficient than conventional PCR, since it only requires access to a constant-temperature apparatus, such as a common laboratory water bath. Third, LAMP is more sensitive than traditional PCR. The LAMP detection limit was 0.74 pg/reaction for *sul1* and 2.6 pg/reaction for *sul2*, which were both 10-fold more sensitive than the corresponding traditional PCR assays. Despite having a higher sensitivity than traditional PCR, the LAMP assays remained specific, and the negative controls used for estimating the specificity were all negative in the LAMP assays. Moreover, after adding HNB, the reaction results could be evaluated by the naked eye, which not only avoids the process of agarose gel electrophoresis that is required with conventional PCR methods, but also reduces the chance of cross-contamination (Liu et al., 2012; Solanki et al., 2013; Yang et al., 2014; Dong et al., 2015b).

Using these LAMP assays, we surveyed the prevalence of *sul* genes among clinical isolates of *S. maltophilia* in three hospitals in Beijing. We isolated 56 SXT-resistant strains and found that they all carried *sul* genes. The results indicated that the carriage of *sul1* and/or *sul2* might be the predominant mechanism of SXT resistance in Beijing, China, as has been reported from other domestic and international surveys (Chang et al., 2007; Toleman et al., 2007; Hu et al., 2011; Chung et al., 2015; Kaur et al., 2015). We also detected both *sul1* and *sul2* in some SXT-susceptible *S. maltophilia* strains. Recent studies also have reported the detection of *sul1* and *sul2* in SXT-susceptible *S. maltophilia* isolates (Chang et al., 2007; Hu et al., 2016). Therefore, the phenotypic breakpoints are not reliable for genotypic identification. In addition, SXT resistance breakpoints are established from a clinical efficacy perspective that also considers the dosage, pharmacokinetics/pharmacodynamics, and clinical trials. SXT treatment might fail for these strains, since resistance could emerge during treatment through the up-regulation of *sul* genes expression.

There are limited antimicrobial options for infection due to *S. maltophilia* because of its extensive resistance to most antibiotics. The occurrence of acquired resistance to SXT mediated by the dissemination of *sul* genes increases the difficulty of selecting an appropriate choice for treatment. To provide epidemiological data for the choice of appropriate antimicrobial agents for the treatment of *sul*-positive *S. maltophilia*, we further tested their susceptibility to 18 clinically used antimicrobials. In previous studies, ceftazidime and ticarcillin/clavulanate were reported as the most effective β-lactam drugs against *S. maltophilia* (Chang et al., 2015). However, only 23% of *sul*-positive strains remained susceptible to ceftazidime in our study, and ceftazidime seemed not to be a good choice due to exhibiting poor *in vitro* activity. Nearly 70% of *sul*-positive *S. maltophilia* strains remained susceptible to ticarcillin/clavulanate, and ticarcillin/clavulanate seemed to be a good choice for the treatment of *sul*-positive strains. However, the recommendation for the use of ticarcillin-clavulanate should be made with caution, since the L1/L2 β-lactamases carried by *S. maltophilia* have strong activity and together can inactivate nearly all β-lactam antibiotics.

New fluoroquinolones, such as moxifloxacin and levofloxacin, have been reported to show good *in vitro* activity and could be alternative therapeutic options against *S. maltophilia* (Valdezate et al., 1999; Nicodemo and Paez, 2007; Looney et al., 2009). Fluoroquinolones are now popular alternatives because of their lesser side effects compared to those of SXT. A clinical comparison of SXT and levofloxacin showed that their overall microbiological cure and clinical success rates showed no statistically significant difference, but the rate of adverse events was significantly lower in the levofloxacin group than in the SXT group (Cho et al., 2014; Wang et al., 2014). However, in our study, nearly 50% of *sul*-positive *S. maltophilia* strains were non-susceptible to moxifloxacin and levofloxacin. Moreover, it has been reported that the overuse of fluoroquinolones worldwide has resulted in a higher resistance rate among many pathogenic bacteria, including *S. maltophilia* (Chang et al., 2014; Pien et al., 2015). Therefore, it would be prudent to use them as initial empirical antibiotics for *sul*-positive strains.

Tetracycline derivatives, such as tigecycline, minocycline, and doxycycline, have been reported to be the most active agents against *S. maltophilia* other than SXT, even in patients with cystic fibrosis. Global surveillance studies have reported that minocycline was found to be significantly more active than other tetracyclines, and the susceptibility rate of *S. maltophilia* to minocycline exceeded 97% across all geographic regions (Gales et al., 2008; Chen et al., 2012; Milne and Gould, 2012; Wu et al., 2012; Chung et al., 2013; Castanheira et al., 2014; Sader et al., 2014). The results of our study agreed with the aforementioned observations, and the tetracycline derivatives we tested, including minocycline, doxycycline, and tigecycline, all showed good *in vitro* activity against clinical isolates of *sul*-negative *S. maltophilia*. Minocycline showed a particularly high *in vitro* activity, with *S. maltophilia* showing a susceptibility rate of 95% against this drug. Tetracycline derivatives might be an appropriate therapeutic option for these *sul*-positive drugs, based on their excellent *in vitro* activities. Ten other agents, including colistin, aztreonam, meropenem, amikacin, azithromycin, fosfomycin, and rifampicin, showed little activity against *sul*-positive *S. maltophilia* strains, and they should not be recommended for use as monotherapy to treat these strains. Epidemiological data might contribute to guiding the choice of an appropriate initial antimicrobial for the treatment of *sul*-positive *S. maltophilia* and reducing the frequency of treatment failures. However, choosing an initial antimicrobial agent by referring to epidemiological data alone is not always adequate, and making more precise treatment choices based on antimicrobial susceptibility testing is encouraged.

## Conclusion

We have developed rapid, simple, and cost-efficient LAMP assays for the detection of the SXT resistance genes *sul1* and *sul2* with high sensitivity and specificity. These assays could contribute to the epidemiological investigation and surveillance of the dissemination of *sul* genes, which could provide epidemiological data to guide the establishment of infection control measures and the choice of initial antimicrobials. Using these methods, 450 non-duplicated *S. maltophilia* strains isolated from three hospitals in Beijing, China were screened, and 72 (16.0%) of these strains tested positive for *sul* genes. These *sul*-positive strains showed poor susceptibility to most clinically used antimicrobials apart from tetracycline derivatives, ticarcillin-clavulanate, moxifloxacin, and levofloxacin. The developed LAMP assays provide a useful tool for the surveillance of *sul* genes among clinical *S. maltophilia* strains, since these assays are more sensitive, faster, and more cost-efficient than the conventional PCR method.

## Author Contributions

YoL and YuL conceived and designed experiments. JZ, YX, WL, WN, CW, and RW executed experiments. JZ, YX, and WN performed and wrote the manuscript. CW and RW helped to edit the manuscript.

## Conflict of Interest Statement

The authors declare that the research was conducted in the absence of any commercial or financial relationships that could be construed as a potential conflict of interest.

## Footnotes

1 http://primerexplorer.jp/e/
